# Molecular profiling of colorectal tumors stratified by the histological tumor-stroma ratio - Increased expression of galectin-1 in tumors with high stromal content

**DOI:** 10.18632/oncotarget.25845

**Published:** 2018-07-31

**Authors:** Tessa P. Sandberg, Jan Oosting, Gabi W. van Pelt, Wilma E. Mesker, Rob A. E. M. Tollenaar, Hans Morreau

**Affiliations:** ^1^ Department of Pathology, Leiden University Medical Centre, Leiden, The Netherlands; ^2^ Department of Surgery, Leiden University Medical Centre, Leiden, The Netherlands

**Keywords:** tumor microenvironment, tumor-stroma ratio, extracellular matrix, cancer-associated fibroblasts, galectin-1

## Abstract

The tumor microenvironment is a dominant determinant of cancer cell behavior. Reactive tumor stroma is associated with poor outcome perspective. The tumor-stroma ratio (TSR) is a strong independent prognostic factor in colorectal cancer and is easily assessed using conventional hematoxylin and eosin (H&E) stained paraffin sections at the invasive margin of the tumor. We aim to understand the biology of the tumor stroma in colorectal cancer by investigating the transcriptomic profiles of tumors classified by the TSR method. The TSR was assessed in a cohort of 71 colorectal cancer patients undergoing surgery without (neo)adjuvant therapy. In the cohort, stroma-high tumors were distinguished from stroma-low tumors at gene expression level in the upregulation of biological pathways related to extracellular matrix (ECM) remodeling and myogenesis. The activated microenvironment in stroma-high tumors overexpressed different types of collagen genes, *THBS2* and *4* as well as *INHBA*, *COX71A* and *LGALS1/galectin-1*. The upregulation of *THBS2*, *COX7A1* and LGALS1/galectin-1. The upregulation of *THBS2*, *COX7A1* and *LGALS1/galectin-1* in stroma-high tumors was validated in The Cancer Genome Atlas. In conclusion, the gene expression data reflects the high stromal content of tumors assessed based on the histological method, the TSR. The composition of the microenvironment suggests an altered proteolysis resulting in ECM remodeling and invasive capacity of tumor cells.

## INTRODUCTION

The tumor microenvironment or tumor stroma is a dominant determinant of cancer cell behavior and disease progression. The tumor stroma constitutes of immune cells, cancer-associated fibroblasts (CAFs), endothelial cells and the extracellular matrix (ECM). During tumor evolution, changes occur in the composition of the tumor stroma. Fibroblasts become activated fibroblasts called CAFs and the overall content of the ECM is remodeled. The ECM of tumors is composed of a complex network of collagen, proteoglycans (such as lumican and versican) and glycoproteins (such as fibronectins, thrombospondins and laminins), locally secreted mainly by CAFs and assembled into a mesh [1]. This network of ECM constituents functions as a scaffold for epithelial and stromal cells and is involved in cell-matrix and cell-cell adhesions which enables tumor cells to migrate. High stromal content, in particular collagen, was associated with a pro-metastatic capacity of cancer cells [2]. CAFs can induce stem cell-like properties and epithelial-to-mesenchymal transition (EMT) in cancer cells [3]. The composition of the tumor microenvironment is an essential aspect of tumor biology [2, 4, 5].

The importance of the tumor microenvironment is also emphasized in the colorectal cancer (CRC) consensus molecular subtypes (CMS), a recent classification developed based on transcriptional profiles. The CMS describes four CRC subtypes, of which the poor-prognosis CMS4 is characterized by high stromal content. CMS4 shows high mesenchymal gene expression, which can be attributed to stromal cells as well as to cancer cells [2, 4–7]. Reactive stroma in solid tumors is associated with poor outcome perspective [5, 8, 9]. We and other research groups have demonstrated that the tumor-stroma ratio (TSR) is a strong independent prognostic factor. The TSR is easily assessed using conventional hematoxylin and eosin (H&E) stained paraffin sections at the invasive margin of the tumor [10–12]. The TSR has been reported in colon cancer as well as in other solid cancer types [10–18]. We aim to understand the biology of the tumor stroma in CRC by investigating the overall transcriptomic profiles of tumors classified by the TSR method using gene expression data. We first compared the quantity of stromal and immune cells based on gene expression in the stroma-low and stroma-high groups using the TSR method. Secondly, we investigated biological pathways differently activated between the stroma-low and stroma-high groups to identify genes of interest. Thirdly, we validated the genes of interest in a second cohort and on protein level.

## RESULTS

### The prognostic value of the tumor-stroma ratio

**Figure 1:**
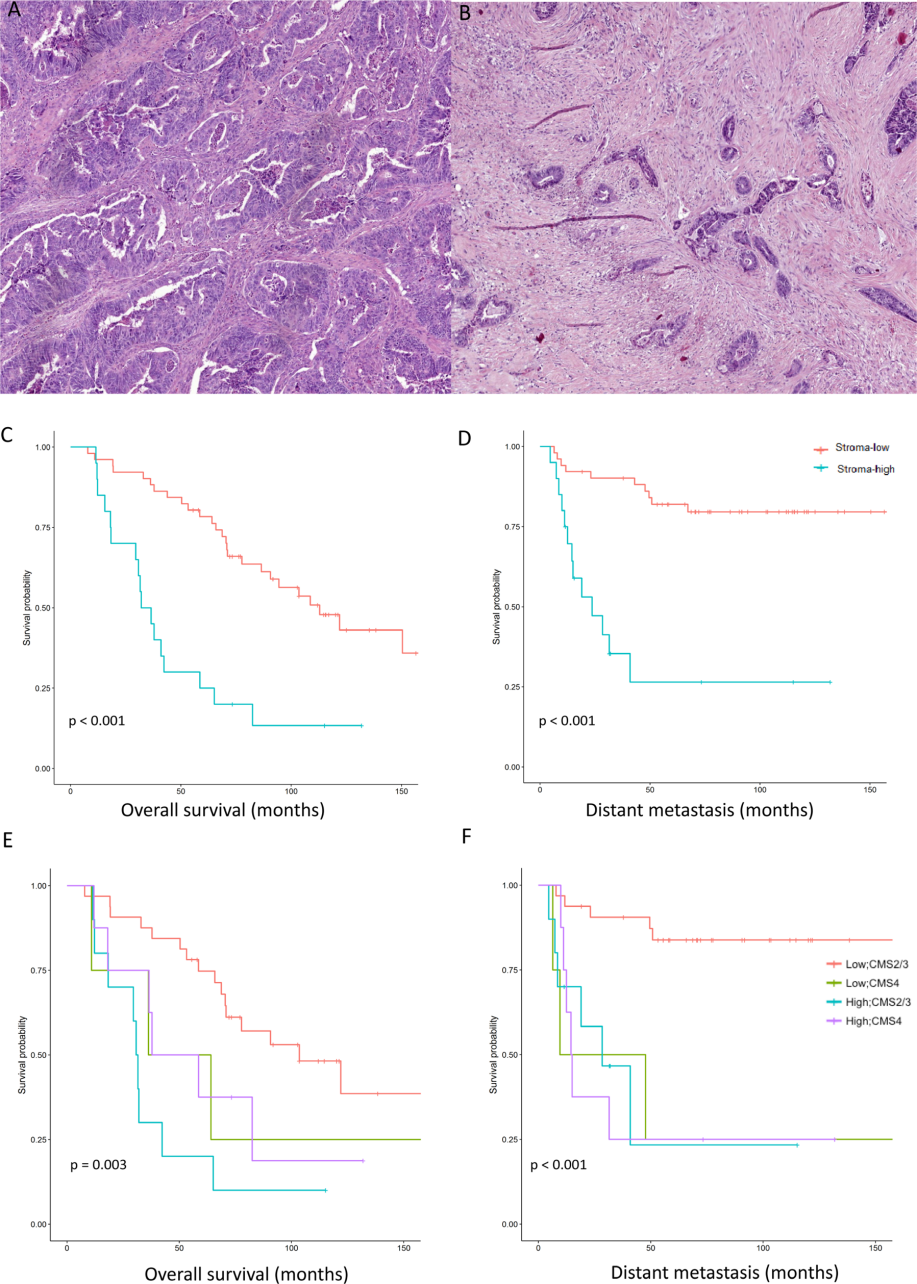
The tumor-stroma ratio identifies patients with poor survival Tumor stroma-low (20% stroma) (**A**) and tumor stroma-high (90% stroma) (**B**) on hematoxylin and eosin sections. Survival curves demonstrating the overall survival (**C**) and the distant metastasis-free survival (**D**) of 71 patients with colorectal cancer stratified by tumor-stroma ratio (TSR) (log-rank test). Survival curves demonstrating the overall survival (**E**) and distant metastasis-free survival (**F**) of 54 patients stratified by both the TSR and CMS classification (log-rank test). CMS1 patients were excluded from the analysis.

A retrospective cohort consisted of 76 sporadic CRC patients undergoing surgery at the Leiden University Medical Centre (LUMC) which were part of a larger cohort [19]. Out of 76 CRC patients, 71 patients were included in the study based on the availability of histological material and of gene expression data. The TSR was scored on H&E sections at the invasive part of the tumor using a microscope (Figure 1A and 1B). Twenty (28.2%) patients belonged to the stroma-high group and 51 (71.8%) to the stroma-low group. The patient characteristics are shown in Table 1. As shown in Figure 1C and 1D, the TSR analysis defined a 5-year overall survival (OS) and distant metastasis-free survival (DMFS) rates of 78.4% and 82.4% in the stroma-low group, and 25% and 35% in the stroma-high group, respectively. The stroma-high group had a significantly worse OS and DMFS rates compared to the stroma-low group (OS *p* = 0.003, HR = 3.76 (1.99–7.09); DMFS *p* = 0.0001, HR = 5.35 (2.40–11.89)). In a multivariate analysis accounting for confounding variables including age, sex and TNM stage, the TSR was an independent predictor for survival (OS *p* = 0.0001, HR = 4.586 (1.96–10.75); DMFS *p* = 0.015, HR = 3.53 (1.273–9.81)) (Table 2). The mesenchymal properties of the CMS4 was shown to be not only attributed to the stromal compartment but also to the epithelial cells. We examined the association between the TSR and the CMS classification (epithelial (CMS2/3) *versus* mesenchymal subtypes (CMS4)) in the LUMC and The Cancer Genome Atlas (TCGA) cohorts. CMS1 patients were excluded. In the LUMC cohort, 17 patients were CMS1. 32/42 stroma-low patients were CMS2/3 and 8/20 stroma-high patients were CMS4 (Table 1). In TCGA, 87/123 stroma-low patients were CMS2/3 and 23/43 stroma-high patients were CMS4 (Supplementary Figure 1A). In both cohorts, the TSR and the CMS classification were associated although with a fair agreement (LUMC: χ^2^ test = 7.714; κ = 0.141; *p* = 0.005; TCGA: χ^2^ test = 7.14, κ = 0.22; *p* = 0.008). A log-rank test was performed in the LUMC cohort categorizing patients by TSR and CMS classification. In the LUMC cohort, stroma-high patients stratified as CMS2/3 or CMS4 did not have a different DMFS nor OS (Figure 1E and 1F). Stroma-low CMS4 patients showed no difference in OS and a worse DMFS compared to stroma-low CMS2/3 patients (OS log-rank test = 1.550, *p* > 0.05; DFMS log-rank test = 11.770, *p* = 0.001; Figure 1E and 1F). No Cox regression model could be fitted due to small numbers in the subgroups.

**Table 1:** Patient characteristics stratified according to tumor-stroma ratio

	Total *N* = 71 (%)	Stroma-Low *N* = 51 (71.8) (%)	Stroma-High *N* = 20 (28.2) (%)
**CMS classification**			
CMS1	17	15	2
CMS2/3	42 (77.8)	32 (88.9)	10 (55.6)
CMS4	12 (22.2)	4 (11.1)	8 (44.4)
**Mean age at surgery**	67.25	65.92	70.85
**Sex**			
Male	33 (46.5)	25 (49)	8 (40)
Female	38 (53.5)	26 (51)	12 (60)
**TNM stage**			
I	10 (14.1)	8 (15.7)	2 (10)
II	39 (54.9)	35 (68.6)	4 (20)
III	22 (31)	8 (15.7)	14 (70)
**MSI status**			
MSS	48 (67.6)	34 (68)	14 (73.7)
MSI-H	21 (29.6)	16 (32)	5 (26.3)
missing	2 (2.8)		
**Location**			
Colon	57 (80.3)	40 (78.5)	17 (85)
Rectum	14 (19.7)	11 (21.6)	3 (15)
**Vital status after 5 years**			
Alive	45 (6.4)	40 (78.4)	5 (25)
Death	26 (36.6)	11 (21.6)	15 (75)

**Table 2:** Univariate and multivariate analyses of overall survival and distant metastasis-free survival stratified according to tumor-stroma ratio in the LUMC cohort of patients with colorectal cancer

		Univariate analysis		Multivariate analysis^a^	
	N events	HR (95% CI)	*P*-value	HR (95% CI)	*P*-value
**Overall survival**	
Stroma-low	51	1	0.003	1	0.0001
Stroma-high	20	3.76 (1.99–7.09)		4.586 (1.96–10.75)	
**Distant metastasis-free survival**						
Stroma-low	51	1	0.0001	1	0.015
Stroma-high	20	5.35 (2.40–11.89)		3.53 (1.273–9.81)	

^a^ Adjusted for age, sex, location of tumor and TNM stage.

### The transcriptomic composition of the microenvironment in stroma-high and stroma-low tumors

**Figure 2:**
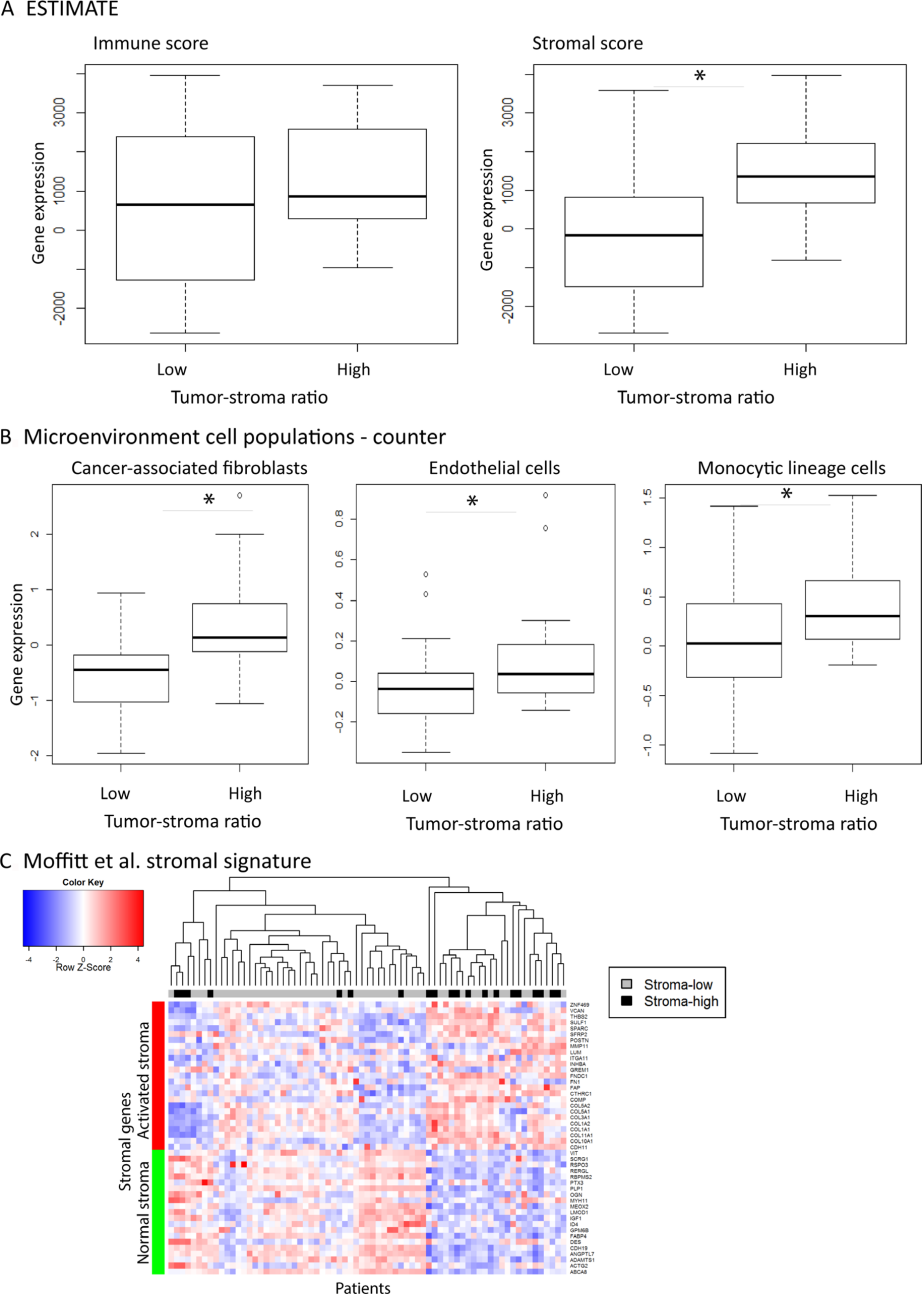
The composition of the stroma in tumors stratified by the tumor-stroma ratio Percentage of stromal and immune infiltration using the ESTIMATE method (**A**), the Microenvironment Cell Populations-counter (**B**) and Moffitt’s stromal signature (**C**) of 71 patients with colorectal cancer stratified by the histological tumor-stroma ratio. mRNA expression of colorectal cancer showed as mean ± S.D with a *p*-value < 0.01 (^*^).

The gene expression profiles of tumors stratified by TSR were investigated. A key challenge in gene expression data analysis is that transcriptomic data is composed of different cell populations including stromal and immune cells. We therefore deconvoluted the samples using three existing computational tools. The ratio of stromal and immune infiltration in the mRNA expression compared to epithelial cells was assessed based on the ESTIMATE gene signatures consisting of 141-stromal and 141-immune genes which were previously shown to be reliable tools [20, 21]. Stroma-high tumors showed a significant increased percentage of stromal infiltration in the mRNA data compared to stroma-low tumors (*t*-test = –4.76, *p* = 2.58^*^10^−5^) while there was no difference in immune infiltration between the stroma-high and -low groups (*t*-test = –1.88, *p* = 0.066; Figure 2A). We further investigated the cell composition of the tumor by assessing the ratio of CAFs, endothelial cells and different immune cell types using the Microenvironment Cell Populations (MCP)-counter [7]. The stroma-high group had a significant increased number of CAFs (*t*-test = –3.91, *p* = 0.0005) and endothelial cells (*t*-test = –2.68, *p* = 0.010) compared to the stroma-low group (Figure 2B). As with the ESTIMATE genes, there was no significant difference in quantity of immune cells between the stroma-high and -low groups, except for cells of the monocytic lineage like macrophages (*t*-test = –2.477, *p* = 0.0203). Moffitt *et al.* developed a stromal signature by virtual microdissection in pancreatic cancer which discriminated between normal and activated stroma [22]. Using this signature, the LUMC cohort was divided into normal (*N* = 44) and activated (*N* = 27) stroma (Figure 2C). Next, we investigated the correlation between the TSR and the MCP-counter CAFs as well as the stromal signature by Moffitt, followed by survival analysis. The LUMC cohort was divided into CAFs low (*N* = 54) and high (*N* = 17) based on MCP-counter CAF markers. The TSR correlated with the MCP-counter CAFs (χ^2^ test = 8.938; *p* = 0.003) and Moffitt’stromal signature (χ^2^ test = 10.830; < = 0.0001). The MCP-counter CAFs high and Moffitt’s activated stromal signature included 3 overlapping genes (*COL1A1*, *COL3A1* and *GREM1*) and correlated with each other (χ^2^ test = 24.477; *p* < 0.0001, Supplementary Figure 2A). Both signatures did not show statistical prognostic value in DMFS although the MCP-counter CAFs high and Moffitt’s activated stroma signature tended to have a decreased DFS (*p* > 0.05, Supplementary Figure 2B and 2C).

### Transcriptomic profiling of the microenvironment in stroma-high and stroma-low tumors

**Figure 3:**
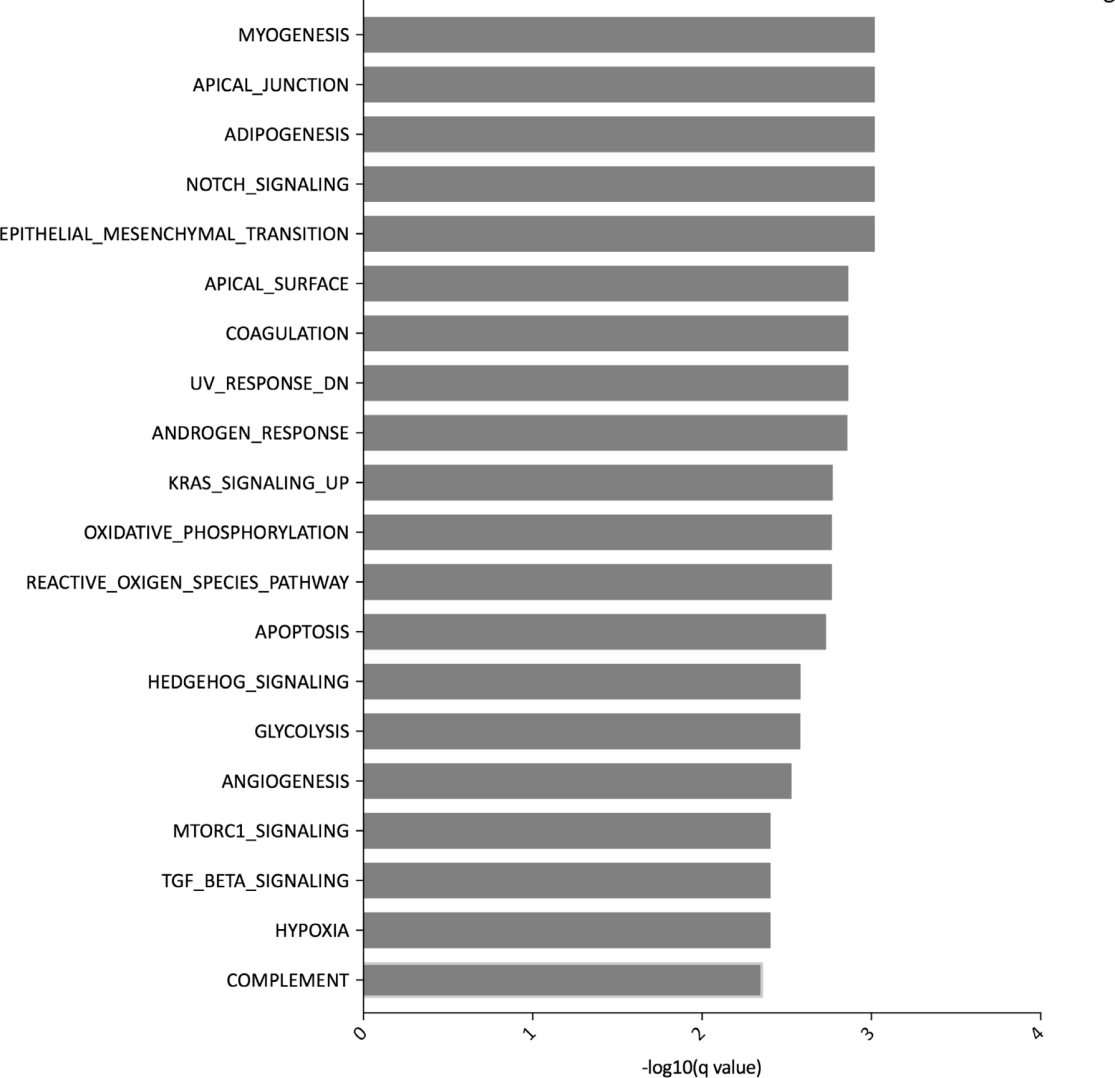
Top 20 pathways of the Hallmark of cancer gene sets differently expressed in tumors stratified according to tumor-stroma ratio

Using the GlobalTest, the transcriptomes of patients stratified according to TSR were significantly different (*p* = 0.0002, Stat = 3.73, SD = 0.359, Covariates = 15923). The publically available Hallmark gene sets on the MSigDB consist of 50 gene sets and were used to explore the difference in transcriptomic pathways between the stroma-low and -high groups [23]. The myogenesis (*p* = 0.0010) and apical junction (*p* = 0.0010) pathways differed most in the two TSR groups. The pathways differing between the TSR groups were mainly related to ECM remodeling, inflammation, metabolism and cell differentiation (Figure 3). To further explore the ECM, we selected transcriptomic pathways related to the ECM and to the function of CAFs (Supplementary Figure 3A). The ECM, focal adhesion and integrin pathways were significantly different between the two TSR groups. Stroma-high tumors expressed high levels of collagen, laminin and integrin subunits. Thorough investigation of the focal adhesion pathway identified two interesting related genes *THBS2* (*p* = 0.0130) and *THBS4* (*p* = 0.0185) coding for thrombonspondin-2 and -4, respectively. Following a network analysis of the two *THBS* genes, *THBS4* was mainly associated with collagen genes and *THBS2* was associated with both collagen genes and ADAM genes (cBioportal; Supplementary Figure 3B; Supplementary Table 1). Within the transforming growth factor- (TGF)β pathway, the genes *INHBA* (*p* = 0.0330), a TGFβ family member, *DCN* (*p* = 0.0007) and *COMP* (*p* = 0.0185) were highly upregulated in the stroma-high group. *COX7A1* gene is known to be expressed by stromal cells [2] and analysis of the myogenesis pathway showed that *COX7A1* coding for a cytochrome C protein was highly expressed in stroma-high compared to stroma-low tumors (*p* = 0.0004). Based on co-expression analysis in the TCGA CRC database, *COX7A1* was highly co-expressed with *LGALS1* coding for galectin-1 (*LGALS1/galectin-1)*, a lectin that is upregulated in the tumor stroma and can inhibit immune response through CD45 protein phosphatase activity (Spearman’s correlation = 0.84). *LGALS1/galectin-1* was also highly expressed in the EMT pathway (*p* = 0.0143). Moreover, *LGALS1/galectin-1* can be expressed by immune cells. As the stroma-low group included 32% tumors with high microsatellite instability (MSI-H), we investigated whether *LGALS1/galectin-1* was differently expressed between MSS and MSI-H tumors, characterized by high immune infiltration. However, there was no difference in *LGALS1/galectin-1* expression (*p* > 0.05). Altogether, stroma-high tumors were characterized by differentiation pathways, changes in metabolism, inflammation and activated ECM compared to stroma-low tumors. The activated microenvironment in stroma-high tumors was characterized by upregulation of different collagen genes, *THBS2* and *4*, *INHBA*, *DCN*, *COMP*, *COX7A1* and *LGALS1/galectin-1*.

### Validation of increased galectin-1, cytochrome C7A1, thrombospondin-2 and -4 expression in the TCGA dataset

Subsequently, we validated the expression of four genes of interest *THBS2, THBS4, COX7A1* and *LGALS1/galectin-1*. As the LUMC cohort was characterized by an increased number of MSI-H patients, we excluded MSI-H patients classified as CMS1 in order to avoid a potential effect of MSI-H status. In total, 166 CRC patients of TCGA had genomic data on cBioportal and H&E tumor sections of the invasive part available on the Cancer Digital Slide Archive website to score TSR. The TSR was scored and showed prognostic value in TCGA (Supplementary Figure 1B). When combining TSR and CMS classification, there was no statistical difference in OS and DMFS (Supplementary Figure 1C). While *THBS4* was not significantly differently expressed (*p* = 0.088), *THBS2*, *COX7A1* and *LGALS1/galectin-1* expression were higher in the stroma-high group compared to the stroma-low group in the TCGA cohort (*THBS2 p* = 0.011; *COX7A1 p* = 0.030; *LGALS1/galectin-1 p* = 0.007).

### Protein expression of galectin-1

Galectin-1 is expressed and released by different cell types and exerts biological functions at different levels of tumor progression [24]. This protein is likely involved in the functional interaction between cancer and stromal cells. Also, research on galectin-1 has mainly focused on its role in tumor and immune cells and not in fibroblasts. We therefore selected galectin-1 for further investigation. We next examined which cell types expressed galectin-1 and whether there was a correlation between galectin-1 expression at protein level and gene expression level. The tumor material of 43 patients of the LUMC cohort was available to perform galectin-1 immunohistochemistry staining.

**Figure 4:**
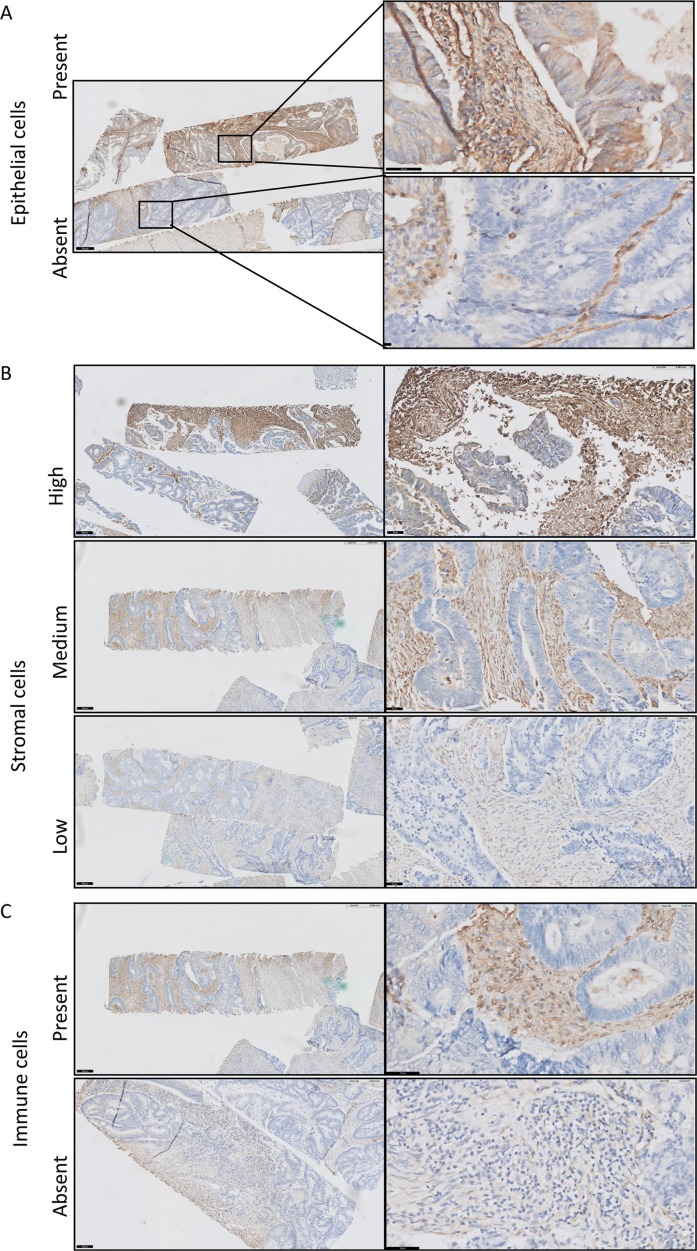
Immunohistochemical staining of galectin-1 in colorectal cancer Galectin-1 is expressed in different compartments of the tumors including in epithelial cells (**A**), in the stromal compartment, likely fibroblasts and endothelial cells (**B**) and on immune cells (**C**).

As demonstrated in Figure 4, galectin-1 was observed in different cell types. Some tumor cells expressed galectin-1 in the cytoplasm at a low staining intensity and percentage (Figure 4A). Galectin-1 was mainly expressed in the stromal compartment (Figure 4B). The staining of galectin-1 was scored in tumor cells and in the stromal compartment. The nuclei and cytoplasm of stromal cells were scored for staining intensity in three categories (low (1.), medium (2.) and high (3.)) and percentage, and the tumor cells were scored for absence (1.) or presence (2.) of staining (Table 3). In this complex pattern, we could not deduce a clear correlation between gene expression and protein level when looking at the score independently and combined (Supplementary Figure 4A). The galectin-1 protein expression in the stromal compartment (including stromal and immune cells) correlated with the TSR. Galectin-1 medium protein expression was associated with high stromal content (χ^2^ test = 10.226; *p* = 0.006; Supplementary Figure 4B). Strikingly, 14 tumors scored as high intensity of galectin-1 protein in the stromal compartment correlated with a good prognosis (DMFS, log-rank = 7.140; *p* = 0.028; Supplementary Figure 4C). This was in contrast to what was expected. Most of these 14 tumors were categorized as stroma-low (13/14) and 5 out of 14 were MSI-H. Based on the immunohistochemistry staining, the high intensity of galectin-1 protein expression was mainly on immune cells (Figure 4C).

**Table 3:** Quantification of galectin-1 expression at protein level ranked by *LGALS1/Galectin-1* gene expression

Patient number	Gene expression	Tumor-stroma ratio^a^	MSI status^b^	Immunohistochemistry score				
				Nuclear intensity^c^	Nuclear per^c^	Cyto intensity^c^	Cyto per^c^	Tumor cells^d^
871	−2.194	1	1	3	20	1	0	2
793	−1.791	1	2	3	50	2	30	2
848	−1.452	1	2	2	90	3	90	1
867	−1.443	1	1	3	50	3	40	1
825	−1.319	1	1	3	90	3	90	1
866	−1.275	1	1	3	80	3	90	1
846	−1.234	1	2	3	50	2	80	2
843	−1.070	1	1	3	10	1	30	2
851	−0.920	1	2	1	70	3	70	1
842	−0.876	1	1	1	90	3	90	1
791	−0.773	1	1	3	30	1	30	1
824	−0.773	2	1	2	70	2	70	2
834	−0.648	1	1	2	30	1	30	1
873	−0.648	1	1	3	0	1	10	2
849	−0.584	1	2	2	0	1	0	1
833	−0.569	2	2	2	0	1	0	1
808	−0.459	1	2	3	90	3	90	1
818	−0.428	2	1	3	50	1	50	1
819	−0.394	2	1	2	70	2	70	1
801	−0.352	1	1	3	90	3	90	2
868	−0.246	1	1	1	50	2	50	1
840	−0.189	1	1	1	0	1	0	1
811	−0.157	1	1	3	0	1	0	1
863	−0.114	2	1	3	30	1	30	1
830	−0.049	1	2	3	80	3	80	1
841	−0.041	1	1	1	100	3	100	1
803	−0.013	1	2	3	0	1	0	2
836	0.065	2	1	3	30	2	30	2
847	0.120	1	1	3	30	1	30	1
829	0.176	1	1	2	80	3	80	1
838	0.193	1	1	1	50	2	70	1
859	0.193	1	1	3	80	3	80	2
820	0.252	2	1	3	90	3	100	1
816	0.286	2	1	3	30	2	30	1
844	0.386	2	2	3	30	1	50	1
860	0.498	1	1	3	10	1	20	1
806	0.539	1	2	3	50	1	50	1
869	0.880	2	2	2	80	2	80	1
855	0.899	1	2	3	70	3	70	1
812	0.936	2	1	3	70	2	70	1
797	1.245	1	2	3	60	1	30	2
845	1.269	2	1	3	0	1	10	2
785	1.313	2	1	3	90	2	90	1

Abbreviations: MSI = microsatellite instability; perc = percentage; cyto = cytoplasma.

^a^Tumor-stroma ratio: 1 = stroma-low, 2 = stroma-high.

^b^MSI status: 1 = microsatellite stable, 2 = MSI-high.

^c^1 = low, 2 = medium, 3 = high expression of galectin-1.

^d^1 = galectin-1 absent, 2 = galectin-1 present.

## DISCUSSION

The TSR showed prognostic value in the LUMC cohort. Patients classified as stroma-high had a worse 5-year DMFS rate (35%). Based on gene expression data, stroma-high tumors were characterized by an increased quantity of CAFs that likely leads to an altered proteolysis and results in ECM remodeling. Supporting this hypothesis is the increased expression of collagen, *THBS* and additional genes involved in the extracellular matrix remodeling in stroma-high compared to stroma-low tumors. Both collagen and *THBS* have been shown to be involved in aggressive behavior of CRC cells [2, 25]. They mediate cell-cell contact and cell-matrix interaction. Our results are in line with previous transcriptomic studies that identified metastatic-associated signatures in multi-cancer types. Key genes contributing to the signature were related to the microenvironment including *THBS2*, *INHBA* and several collagen genes [26, 27].

In addition, stroma-high tumors were associated with an increased mRNA expression of *LGALS1/galectin-1* in two cohorts. Galectin-1 is a galactoside-binding protein which localizes both intra- and extracellularly and has a wide range of biological functions. In tumors, intracellular galectin-1 modulates cell signalling through protein-protein binding. For instance, galectin-1 binds to H-RAS which drives tumor transformation (Supplementary Figure 2B) [28]. The carbohydrate-recognition domain of extracellular galectin-1 can bind to carbohydrates located at the cell surface of cancer and stromal cells. These interactions result in modelling cell adhesion and migration of the target cell. Galectin-1 can also induce an apoptotic response in immune cells by binding for instance CD45. Furthermore, galectin-1 interacts with glycoproteins of the ECM such as laminin, thrombospondin, vitronectin, fibronectin and osteopontin, which were highly expressed in stroma-high tumors [29, 30]. Previous studies demonstrated in different tumor types, including colon cancer, an association between high galectin-1 expression and poor prognosis [24, 31–34].

Most studies investigated the expression of galectin-1 in cancer epithelial and immune cells while we found an increased expression of galectin-1 in stroma-high tumors. Therefore, we further investigated the localization of galectin-1 in the tumor at the protein level. The present study showed that galectin-1 was expressed by CAFs, immune, endothelial and tumor cells at different intensities. In this complex pattern, we could not deduce a clear correlation of *LGALS1/galectin-1* at gene expression level and protein level, which was also observed in a previous study [35]. The immunohistochemical results of the present study identified tumors of which the immune cells showed particular upregulated galectin-1 expression and a really good DMFS rate. We hypothesize that tumors with high galectin-1 expression specifically on immune cells reflects an antitumor immune response. During tumor progression, stromal cells, in particular CAFs, increase galectin-1 secretion which suppresses the immune response and is involved in tumor invasion [36]. This suggests that upregulated galectin-1 expression in stroma-high tumors provides a microenvironment characterized by immune suppressive response resulting in invasive tumor cells. Furthermore, the question remains what drives the activation of CAFs. TGFβ is known to activate CAFs and it was shown that this growth factor induced galectin-1 expression in fibroblasts [37, 38]. However, the biological mechanism of galectin-1 remains complex. The role of galectin-1 is likely a balance of different factors including the ECM composition, cellular localization and the cell type, among others. Further research is needed to investigate the role of galectin-1 in the complex interaction between cancer and stromal cells leading to the aggressive behavior of cancer cells.

The TSR was scored at the most invasive part of the tumor while the mRNA was isolated from the tumor bulk. Strikingly, the gene expression data reflects high stromal score, including an increased number of CAFs, endothelial cells and cells of the monocytic lineage. No difference in immune cells was found between the stroma-high and -low tumors. Previous studies showed that CAFs drive immune evasion through for instance TGFβ [39]. One study found in invasive ductal breast cancer an inverse relationship between high stromal content measured by the TSR and macrophage and T-cell infiltration [40]. Another study investigated the association between TSR and inflammatory response in CRC. The authors did not find any association between TSR and T cell infiltration and an inverse association borderline significant between TSR and immune cell infiltration measured on H&E sections [16]. Most interestingly, they later found that combining immune infiltration and TSR added prognostic value [41].

A first limitation of this study is that the LUMC cohort comprised an increased number (29.5%) of MSI-H patients, which is not representative with the reality (15%) [42]. Secondly, galectin-1 immunohistochemistry was performed on perpendicular tumor punches where the orientation of the tumor was unknown. It was not possible to assess the level of galectin-1 expression in the standardized manner at the most invasive part of the tumor, which is the region expected to have an increased amount of CAFs and remodeled ECM [43].

Given the current high costs of transcriptomic data, standard pathological assessment relies heavily on microscopy. Therefore, it is of interest to use a microscopy-based method to select patients which will benefit or not from (targeted) therapy. The TSR can be used to identify patients with increased stromal infiltration and a poor prognosis. Previous research has shown that the activation level of stromal cells is associated with prognosis [4, 8]. Tumors classified as stroma-high and CMS4 overlapped to a certain extent. Both methods have their limitations, which are likely related to the methodology and the tumor heterogeneity. In the era of personalized medicine, a main goal is to increase the predictive value of subsets of patients. CMS4 patients are known to respond poorly to treatment [6, 44, 45]. Once beneficial treatment for colorectal tumors with high stromal content will be available in the clinic, an easy to use stratification method will be necessary such as the TSR.

## MATERIALS AND METHODS

### Cohorts

A retrospective cohort consisted of 76 sporadic CRC patients treated at the LUMC between 1991 and 2005, and diagnosed as TNM stages I, II and III. The LUMC cohort was previously analyzed as part of a larger cohort [19]. Patients underwent surgery without any (neo)adjuvant therapy. MSI-H status had been determined for this cohort as described previously [46]. All samples were handled according to the National Ethical Guidelines. The gene expression data of CRC patients of TCGA were used as a validation cohort [47].

### Tumor-stroma ratio score

Patient material was fixed in formalin and embedded in paraffin and consisted of 5 µm H&E-stained sections from the most invasive part of the primary tumor. On the same H&E section, two investigators, independently, selected and estimated the region with the highest stroma percentage in a blinded manner using a 2.5× or 10× microscopic field. A ×10 objective microscopic field was scored where tumor cells were present at two opposite borders of the image field (example in Figure 1A and 1B). Scoring percentages were given in 10 fold percentage per image field and the final score was assessed in the field with the highest stroma percentage. Tumors with less than or equal to 50% of stroma were considered stroma-low and tumors with greater than 50% of stroma were considered stroma-high.

The TSR of patients of the TCGA was determined using H&E sections available online on the Cancer Digital Slide Archive (http://cancer.digitalslidearchive.net/). Only pathological sections estimated to be the most invasive part of the tumor were used. A zoomed in area was used to score the TSR in a similar manner as described above.

### mRNA expression array and analysis

The mRNA of the LUMC cohort was previously isolated from fresh frozen tissue and hybridized to a customized Agendia 44 K oligonucleotide array as described elsewhere [19]. The quantity of stromal and immune cells were estimated using the online R package ESTIMATE [20], the MCP-counter v1.1.0 [48] and Moffitt’s stromal signature [22]. The LUMC cohort was divided into high and low fibroblast expression based on the fibroblast markers of the MCP-counter using a cut-off at the 3rd quartile. Based on 46 out of the 48 genes of Moffitt’s stromal signature, the LUMC cohort was clustered using correlation as a distance metric with average linkage.

To identify gene sets differently expressed between the two TSR groups, publically available databases were selected from MSigDB website. The statistical analysis of the mRNA expression data was done using the Global test as well as to further investigate genes differently expressed within gene sets [49, 50]. The global tests were followed by multiple testing correction using False Discovery Rate (FDR) in case of comparing the gene sets and the inheritance procedure in case of genes within a gene set [51]. Finally, patients of the LUMC cohort were classified for CMS using the R package described previously and patients of the TCGA were previously classified in CMS [6]. mRNA data of patients of the TCGA cohort was downloaded from http://www.cbioportal.org/ where a network analysis was performed for the genes of interest.

### Immunohistochemistry

Galectin-1 immunohistochemistry was performed on previously punched formalin-fixed, paraffin-embedded tumors of the LUMC cohort to investigate the level of galectin-1 at protein level. Punches were perpendicularly re-embedded in paraffin. 4 µm sections were cut and dried overnight at 37° C. On the day of the immunohistochemistry, sections were deparaffinized, rehydrated and underwent a 20 minutes incubation in a 0.3% hydrogen peroxide solution (Millipore). The sections underwent antigen retrieval by heating 10 min at 95° C in pH low Target Retrieval Solution (Dako) and allowed to cool down. Unspecific binding sites were blocked with 5% goat serum (Dako) for 15 minutes. Monoclonal primary antibody against endogenous galectin-1 (1:400, D608T, Cell Signaling) was applied overnight. The following day, secondary HRP labelled antibody anti-rabbit (Dako EnVision+) was applied for 30 minutes. Antigen-antibody complexes were visualized using 3,3′-diaminobenzidine (DAB)+ Substrate-Chromogen System (Dako). Finally, sections were counterstained with hematoxylin and mounted in Pertex. In addition to the galectin-1 staining, a sequential section was stained with H&E to identify different cell type and tissue structure.

### Survival and statistical analysis

Statistical analyses were performed using R version 3.3.0. OS time was defined as the time period between surgery and death or end of follow-up. DMFS time was defined as the time period between surgery and metastasis or end of follow-up [52]. Univariate and multivariate Cox regression analyses were performed to test the differences in OS and DMFS between patients stratified according to TSR. Covariates entered in the model were age, sex, TNM classification and location of the tumor (colon *versus* rectum). Kaplan–Meier curves and log-rank tests were performed to compare the survival probabilities between groups. Student *t*-tests were performed to test the transcriptomic differences in composition of the microenvironment (ESTIMATE and MCP-counter) between stroma-low and -high groups as well as in four genes of interests in TCGA. A Pearson’s chi-squared test (χ^2^ test) was performed to evaluate the association between the TSR and subgroups including CMS subtypes (epithelial *versus* mesenchymal), the CAFs-low and -high based on MCP-counter and the normal and activated stroma using Moffitt’s stromal signature. The level of significance was set at *p* < 0.05.

## SUPPLEMENTARY MATERIALS FIGURES AND TABLE





## Abbreviations

CAFs: Cancer-associated fibroblasts
CMS: Consensus Molecular Subtypes
CRC: Colorectal cancer
DMFS: Distant metastasis-free survival
ECM: Extracellular matrix
EMT: Epithelial-mesenchymal transition
H&E: Hematoxylin and eosin
LUMC: Leiden University Medical Centre
*LGALS1/galectin-1*: Galectin-1 gene
MCP-counter: Microenvironment Cell Populations -counter
OS: Overall survival
TCGA: The Cancer Genome Atlas
TGFβ: Transforming growth factor-β
TSR: Tumor-stroma ratio
