# Erratum: Molecular profiling of colorectal tumors stratified by the histological tumor-stroma ratio - Increased expression of galectin-1 in tumors with high stromal content

**DOI:** 10.18632/oncotarget.26820

**Published:** 2019-03-22

**Authors:** Tessa P. Sandberg, Jan Oosting, Gabi W. van Pelt, Wilma E. Mesker, Rob A.E.M. Tollenaar, Hans Morreau

**Affiliations:** ^1^ Department of Pathology, Leiden University Medical Centre, Leiden, The Netherlands; ^2^ Department of Surgery, Leiden University Medical Centre, Leiden, The Netherlands

**This article has been corrected:** During production, the text ‘The upregulation of THBS2, COX7A1 and LGALS1/galectin-1’ was placed twice in the Abstract section. The duplicate line has been deleted and the proper Abstract is shown below.

**ABSTRACT**

**The tumor microenvironment is a dominant determinant of cancer cell behavior. Reactive tumor stroma is associated with poor outcome perspective. The tumor-stroma ratio (TSR) is a strong independent prognostic factor in colorectal cancer and is easily assessed using conventional hematoxylin and eosin (H&E) stained paraffin sections at the invasive margin of the tumor. We aim to understand the biology of the tumor stroma in colorectal cancer by investigating the transcriptomic profiles of tumors classified by the TSR method. The TSR was assessed in a cohort of 71 colorectal cancer patients undergoing surgery without (neo)adjuvant therapy. In the cohort, stroma-high tumors were distinguished from stroma-low tumors at gene expression level in the upregulation of biological pathways related to extracellular matrix (ECM) remodeling and myogenesis. The activated microenvironment in stroma-high tumors overexpressed different types of collagen genes, THBS2 and 4 as well as *INHBA*, *COX71A* and *LGALS1/galectin-1*. The upregulation of *THBS2*, *COX7A1* and *LGALS1/galectin-1* in stroma-high tumors was validated in The Cancer Genome Atlas. In conclusion, the gene expression data reflects the high stromal content of tumors assessed based on the histological method, the TSR. The composition of the microenvironment suggests an altered proteolysis resulting in ECM remodeling and invasive capacity of tumor cells.**

Original article: Oncotarget. 2018; 9:31502-31515. https://doi.org/10.18632/oncotarget.25845

