# Supplementary material for: Molecular profiling of colorectal tumors stratified by the histological tumor-stroma ratio - Increased expression of galectin-1 in tumors with high stromal content
Source: Oncotarget. 2018 Jul 31;9(59):31502–15. doi: 10.18632/oncotarget.25845 (PMC6101138; doi:10.18632/oncotarget.25845)
Supplement: Supplementary file 1 [file oncotarget-09-31502-s001.pdf]

Molecular profiling of colorectal tumors stratified by the histological tumor-stroma ratio - Increased expression of galectin-1 in tumors with high stromal content

SUPPLEMENTARY MATERIALS

A Tumor-stroma ratio and CMS classification

	Tumor-stroma ratio		Total
	Stroma-low	Stroma-high	
**CMS classification**			
CMS2/3	87	20	107
CMS4	36	23	59
Total	123	43	166

B Tumor-stroma ratio

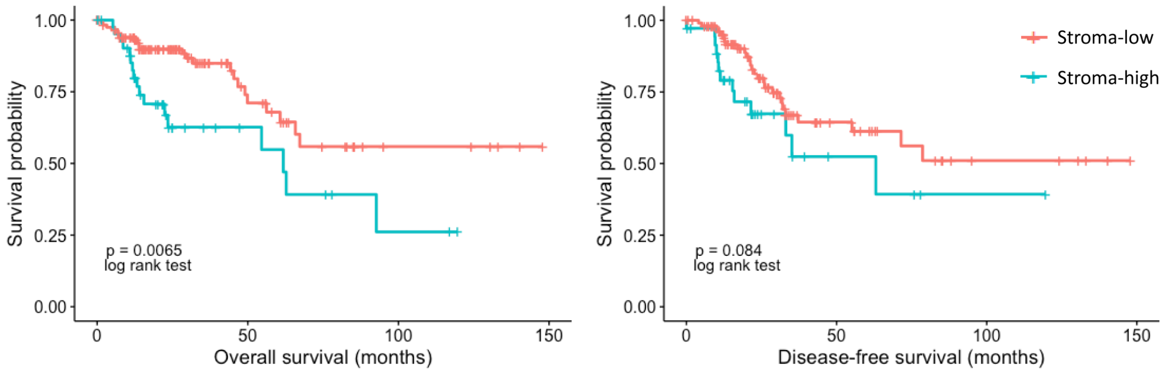

C Tumor-stroma ratio and CMS classification

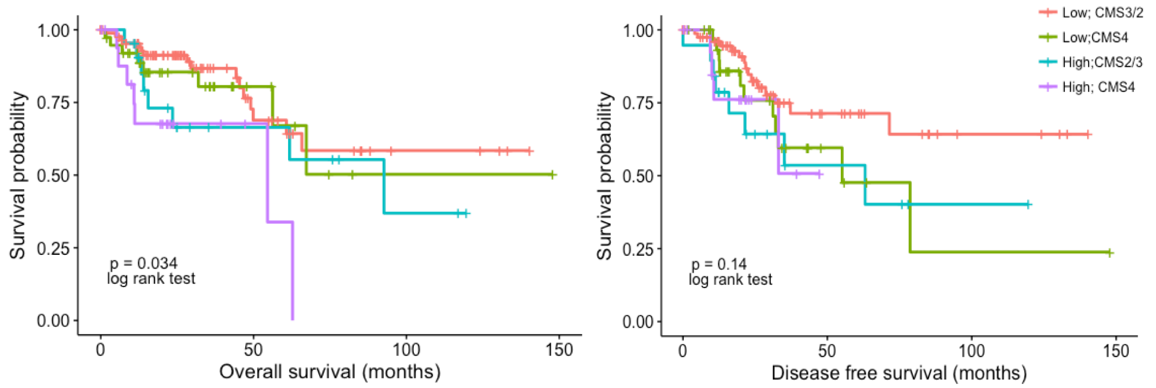

Supplementary Figure 1: Classification and survival curves of 166 patients of The Cancer Genome Atlas stratified by the tumor-stroma ratio and CMS classification. Cross table of patients classified by the histological tumor-stroma ratio and the CMS classification based on gene expression data (**A**). Survival curves demonstrating the overall survival and the disease-free survival of 166 colorectal cancer patients of TCGA stratified by the tumor-stroma ratio (TSR) (**B**) and by the combination of the TSR and CMS classification (**C**) (log-rank test).

A Association between tumor-stroma ratio, MCP-counter CAF and Moffitt's stromal signature

	Tumor-stroma ratio		Moffitt's stromal signature		Total
	Low	High	Normal	Activated	
MCP-counter					
Low	43	10	43	10	53
High	8	10	3	15	18
Total	51	20	46	25	
Moffitt's stromal signature					
Normal	39	7			
Activated	12	13			

B MCP-counter cancer-associated fibroblasts

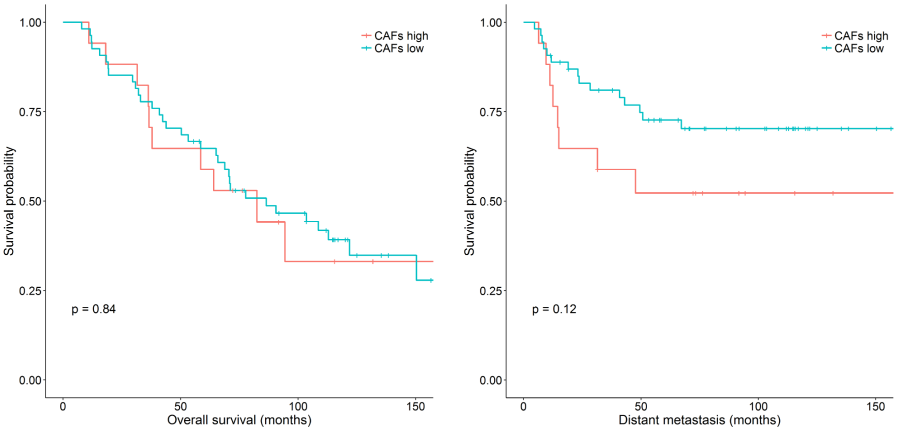

C Moffitt stromal signature

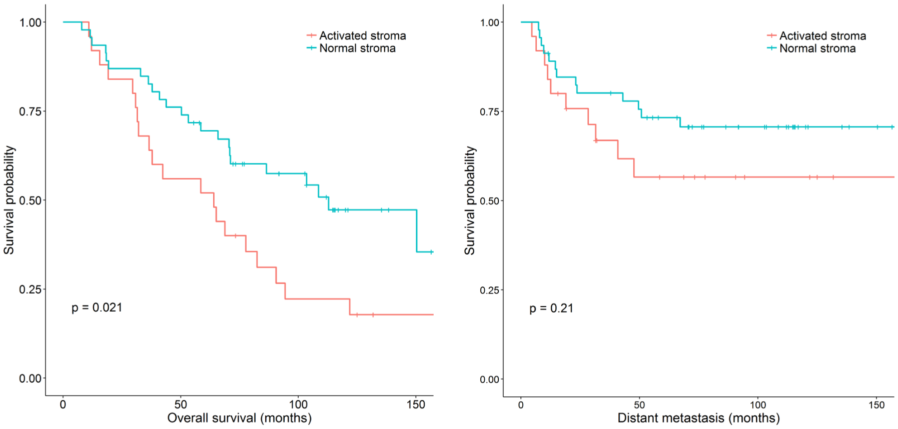

Supplementary Figure 2: Association and survival of tumor-stroma ratio, MCP-counter CAFs and Moffitt's stromal signature. Cross table of patients classified by the histological tumor-stroma ratio and the transcriptomic MCP-counter CAFs and Moffitt's stromal signature (**A**). Survival curves demonstrating the overall survival and the distant metastasis-free survival of 71 colorectal cancer patient of the LUMC cohort. The patients were stratified using the MCP-counter CAFs (**B**) and Moffitt's stromal signature (**C**) (log-rank test).

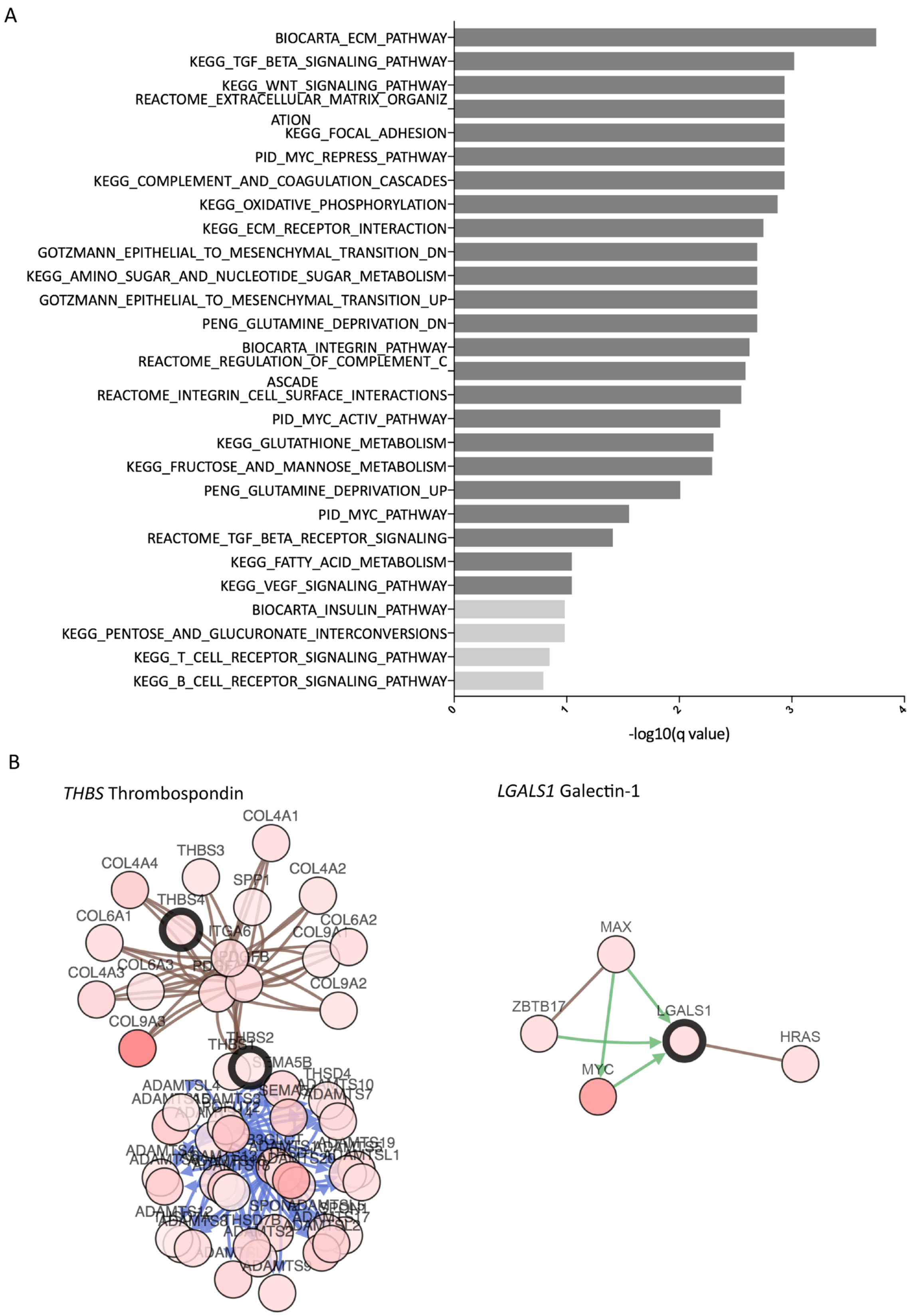

Supplementary Figure 3: Selection of transcriptomic pathways related to the extracellular matrix and to cancer-associated fibroblasts. Gene sets differently expressed in tumors stratified according to tumor-stroma ratio (**A**). Bars in grey are significantly different, bars in light grey are not significantly different between the two groups. Network predicting the association of thrombospondin-2 and -4, and galectin-1 with other proteins (**B**). Presence of protein in complex with (brown line), controls expression of (green line), controls state of change (blue line).

A

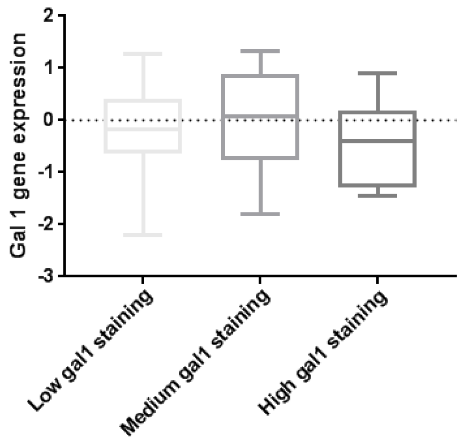

B

	Galectin-1 cytoplasma intensity - immunohistochemistry			Total
	Low	Medium	High	
**Tumor-stroma ratio**				
Stroma-low	13	4	13	30
Stroma-high	5	7	1	13
Total	18	11	14	43

C

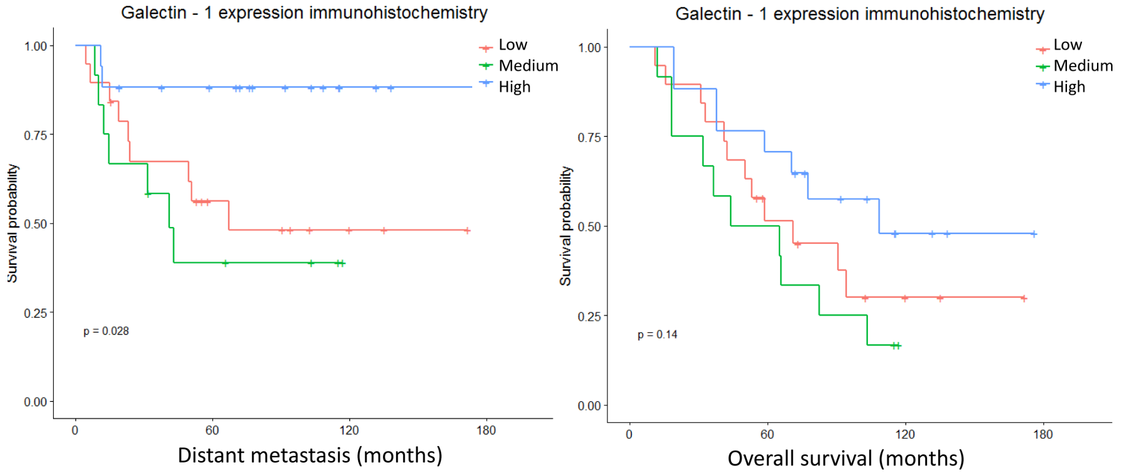

Supplementary Figure 4: Survival analysis of 43 patients of the LUMC cohort stratified by the galectin-1 staining intensity in the stromal compartment.

Supplementary Table 1: Genes differently expressed between stroma-low and -high tumours within each pathway. See Supplementary_Table_1
